# Implementation of a value-based approach for older people who have suffered an acute myocardial infarction: study protocol

**DOI:** 10.3389/fpubh.2024.1518469

**Published:** 2025-01-03

**Authors:** Denis Juraga, Tomislav Rukavina, Mihaela Marinović Glavić, Lovorka Bilajac, Aleksandar Racz, Esmee L. S. Bally, Oscar Zanutto, Tamara Alhambra-Borrás, Maite Ferrando, Alen Subotić, Hein Raat, Vanja Vasiljev

**Affiliations:** ^1^Department of Social Medicine and Epidemiology, Faculty of Medicine, University of Rijeka, Rijeka, Croatia; ^2^Teaching Institute of Public Health of Primorje-Gorski Kotar County, Rijeka, Croatia; ^3^Department of Public Health, Faculty of Health Studies, University of Rijeka, Rijeka, Croatia; ^4^University of Applied Health Sciences, Zagreb, Croatia; ^5^Department of Public Health, Erasmus University Medical Centre, Rotterdam, Netherlands; ^6^European Project Office Department, Istituto Per Servizi Di Ricovero E Assistenza Agli Anziani (Institute for Hospitalization and Care for the Elderly), Treviso, Italy; ^7^Polibienestar Research Institute, University of Valencia, Valencia, Spain; ^8^R&D+I Consultancy, Kveloce I+D+i (Senior Europa SL), Valencia, Spain; ^9^Department of Emergency Medicine, Velika Gorica, Croatia

**Keywords:** elderly, myocardial infarction, decision making, shared, value-based health care, health care professionals

## Abstract

**Introduction:**

Due to the rapid aging of the global population, new approaches are required to improve the quality of life of older people and to reduce healthcare system expenditures. One of the approaches that can be used is value-based healthcare. This article describes a value-based solution for older people who have suffered a myocardial infarction.

**Methods:**

This solution combines the work of healthcare professionals and informal caregivers and the use of modern and user-friendly technologies to support the achievement of patients’ values. Patients older than 65 years who have suffered a myocardial infarction will be divided into control and intervention groups within a pre-post-controlled design research study. Members of the intervention group will be provided with a personalized plan developed by healthcare professionals and based on the results from the baseline questionnaire.

**Discussion:**

Two ValueCare digital solution components will be developed: a mobile application for the participants and a web platform for the professionals, researchers, and informal caregivers. Together with smartwatches, which will track important health aspects, and applications, this approach would enable older people to improve their health through correct lifestyle choices and their professional and informal caregivers to track their progress. With the use of the described technology and the multidisciplinary approach, the unmet needs and values of participants could be achieved. Using this approach, it could be possible to reduce overall healthcare expenses through the active involvement of both older people and their informal caregivers through a shared decision-making process with healthcare professionals.

**Clinical trial registration:**

The ISRCTN registry number is 25089186. The date of trial registration is 16/11/2021.

## 1 Introduction

Demographic changes in the world’s population pose a major challenge to social and healthcare systems. In 2020, there were more adults over the age of 60 than children under the age of five (1). Aging is characterized by numerous challenges, such as chronic diseases, risk of falls, frailty, and mental health disorders (1). Multimorbidity in old age, characterized by more than one chronic disease, is also widespread (2, 3), and some studies indicate that it can reach 51.1%, leading to increased healthcare utilization and thus increased costs (2). Against this backdrop, the United Nations (UN) Decade of Healthy Aging (2021–2030) was declared the top priority of the decade, following the Plan of Action adoption on August 3, 2020, at the 73rd World Health Assembly (4). To promote healthy aging, healthcare systems should focus more on the outcomes of care than on the volume of services provided while implementing person-centered and coordinated care (5). This can be achieved by shifting the focus from the cost of a treatment to the value it provides to the patient, i.e., value-based healthcare (VBHC). This novel approach has attracted much attention in recent years (6). The VBHC approach considers the health outcomes relevant to the patient and the costs required to achieve these outcomes and aims to improve patient health in an efficient manner (7). VBHC ideally reduces both healthcare and patient spending and improves the overall health of the population (8). Reducing overall healthcare expenditure (by reducing hospitalization and medical emergencies) is of particular importance for aging societies. It should be noted that there are different methods and tools available for achieving successful VBHC (6). To improve the outcomes of care, different approaches, such as providing mobile healthcare applications that enable patients and healthcare providers to monitor goals and achievements, are available worldwide. In addition, healthcare providers can also make data-driven decisions based on the collected data and the needs of patients. One of the tools that has gained popularity with the advancement of mobile technology is the development and design of mobile healthcare applications based on the values and opinions of end users (9).

According to Eurostat, cardiovascular diseases, including myocardial infarction, are the leading cause of death in the European Union (10). Myocardial infarction (MI) is an acute condition that occurs when blood flow in the heart muscle is reduced or blocked (11). The infarction may go unnoticed, or symptoms may occur, but it can also be mistaken for another condition (12). The INTERHEART study revealed that the main risk factors for developing MI, such as smoking, obesity, diabetes, and unhealthy diet, are modifiable (13). With appropriate management of risk factors following MI incidents, patients’ health and well-being can improve if changes are introduced as a continuous, value-based process enhanced with digital technology so that the patient is not overwhelmed (14, 15). With the combination of targeted lifestyle changes and a holistic approach, as well as the addition of personalized technology and outcome-based care, it is possible to maintain health and prevent disease progression (16, 17).

The integration of lifestyle medicine, personalized integrated technology and outcome-based care will be tested in seven large-scale pilot projects in the ValueCare project (Value-Based Methodology for Integrated Care Supported by Information and Communication Technologies, Grant Agreement No. 875215) (18). The aim of this study protocol is to introduce a value-based approach, supported by a digital solution at the pilot site in Rijeka, Croatia, in a population of patients who have suffered an acute myocardial infarction and have completed the rehabilitation process. This could improve their quality of life (and that of their families), reduce the burden on healthcare professionals and improve the sustainability of the health and social care system.

## 2 Methods and analysis

### 2.1 Design of the study

The ValueCare methodology will be based on an interactive process that focuses on the needs and requirements of the end users as well as ethical and data protection aspects. The goal and aim of the ValueCare study are to develop a new, outcome-based model of integrated social and healthcare supported by a digital solution that promotes the health and social goals of older citizens, supports informal caregivers, and improves the working conditions of professionals. The ValueCare study work plan will be divided into eight work packages to implement and achieve objectives while optimizing time, resources, and quality. The vision of integrated, value-based care will be supported by a robust, secure, and scalable digital solution that will be tested and evaluated at pilot sites in Europe according to a methodology that will be developed by the project partners together with end users within a predefined protocol for co-design activities. The seven pilot sites included, in alphabetical order, will be Athens, Greece; Coimbra, Portugal; Cork/Kerry, Ireland; Rijeka, Croatia; Rotterdam, The Netherlands; Treviso, Italy; and Valencia, Spain (18).

The integrated care approach will be developed to shift care away from acute episodic hospital care to community-based, planned, proactive and coordinated care. The ValueCare study will use the International Consortium for Health Outcomes Measurement (ICHOM) dataset for older people as well as for specific conditions (e.g., stroke, diabetes) and will contribute to their further development by analyzing the outcome parameters together with the global ICHOM working group to determine if they are complete and still appropriate (18, 19).

The first step of the ValueCare study will be to carry out the co-design activities. This iterative process will enable a wide range of included stakeholders to bring creative contribution into a dialogue. The co-design approach will bring participants the opportunity to identify common challenges and values and discuss what is the best way to address them. The role of the participants will be therefore crucial as the outcomes of the co-design discussion will address the challenge(s) identified by the participants themselves. It will be of utmost importance that participants play an active role when taking part in co-design activities, which will rely on a mutual collaboration between them.

The co-design approach, which will be organized in three time points (three rounds) before the start of the intervention, will involve different groups of stakeholders, including professionals and patients as well as their informal caregivers. While health and social care professionals, IT experts and managers (providers/supply-side) will bring their expertise to the discussion, and citizens and their informal caregivers (end-users/demand-side) will bring their empirical knowledge and experience, becoming pivotal element of the discussion and to the future development of the value-based concept and the whole ValueCare digital solution. The first round will consist of focus groups with stakeholders and their opinion regarding the current state of care, and their actual needs, the second round will be dedicated to the concept of the ValueCare solution, while the third round will be focused on the feedback for the improvement of the digital solution, after the testing phase. In the case of older people that suffered MI, the main target group of Rijeka pilot site, this approach will bring the opportunity to better understand older people’s daily lives, needs, values, experiences, and functional abilities. This will involve their cooperation and interactivity in the development of the study methodology, which will be based on the needs of end users and developed together with the relevant stakeholders (health and social care professionals, informal caregivers, IT experts, managers, and exploitation stakeholders). In this context, the main objective of co-design activities will be to give older people, as well as other stakeholders involved, the opportunity to influence their present and future care pathways and to personalize the services they receive. The co-design activities will consist of three main approaches: targeted semi-structured interviews, focus groups and a national seminar, as they are the main tools used in co-design implementation within healthcare systems for digital solution deployment (18, 20–23). During the organization and conduction of these three approaches, the initial suggestions on how the ValueCare concept should be and how the supportive IT solution should be integrated to respond to the needs of the target groups will be gathered as well as the feedback on the ValueCare approach presented (ValueCare concept and the whole digital solution) and the ValueCare standards, person-centered approach and interoperability of the interdisciplinary team involved in the future intervention phase. The key principle of co-design will be the central role of end users and stakeholders and their involvement and engagement throughout the process. In this context, the intervention of ValueCare will improve the quality of life, independence, and satisfaction of older people through the development of digital solutions, which will enable them to remain independent. As of the main outcome based on the results of the co-design activities, the ValueCare digital solution will be developed. It will provide health information to health and social care professionals so that they could interpret this information, make value-based decisions in real time and personalize the care services provided. The digital solution that will be developed as part of the ValueCare study will consist of two main segments: the Vida24 ValueCare web application and the ValueCare mobile application. The Vida24 ValueCare web application will be the main dashboard for health and social care professionals, researchers and informal caregivers. It will be used by a multidisciplinary team (cardiologists, community patronage nurses and physiotherapists) to develop personalized, value-based care plans. Informal caregivers will mainly use the dashboard to monitor the progress of their family members, i.e., older individuals. The most important function of the ValueCare mobile application will be the Virtual Coach, a virtual chat bot that will contain conversation patterns developed specifically for each participant based on their values and needs.

**Figure 1:**
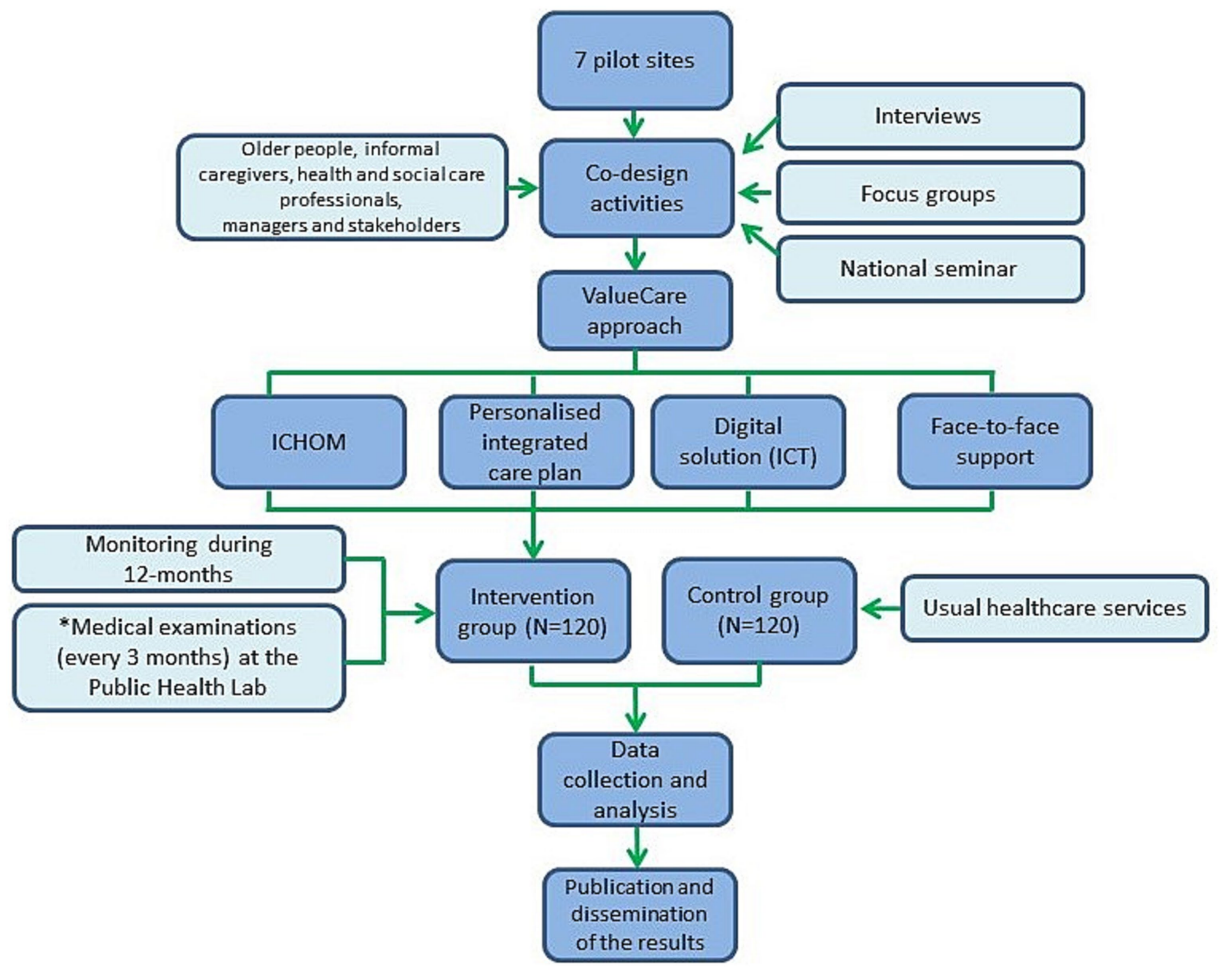
Design of the ValueCare study at the Rijeka pilot site.

When the activities of the previously mentioned co-design and the testing of the digital solution will be completed, the researchers, together with health professionals and other stakeholders, will start the inclusion of the participants. The design of the ValueCare study for the Rijeka pilot site is summarized in Figure 1.

### 2.2 Selection and inclusion of the participants

The participants in this study will consist of older people, informal caregivers, and health (general practitioners, general practice nurses, community patronage nurses, cardiologists and physiotherapists) and social care professionals.

The criteria for inclusion in the older group will be individuals aged 65 years and older, of both sexes, who had suffered MI and had completed the rehabilitation process. They should live in their own household in the city of Rijeka or its surroundings. The exclusion criteria will be dementia, terminal illness, inability to participate in the project for 20 months and institutionalization. These criteria will be agreed upon within the ValueCare project consortium since it is a community-based intervention in an urban environment. Participants will be identified and selected in cooperation with health professionals (cardiologists, nurses) from the Clinic for Rehabilitation, Treatment and Prevention of Cardiovascular Diseases, Thalassotherapia Opatija (24) and Community Health Centre of Primorje-Gorski Kotar County (25). Participants who will meet the inclusion criteria and have successfully completed the rehabilitation process after an MI will be introduced to the ValueCare approach a few days before they are discharged from Thalassotherapia Opatija so that they can familiarize themselves with the project and the study. After the presentation of the ValueCare project, interested participants will formally join the study. A total of 240 older people will participate in the study. They will be divided into two groups. Half of them (120 people) will participate in the intervention phase for 12 months and receive enhanced care supported by the ValueCare digital solution, while the remaining 120 participants will form the control group and receive usual care. Since we assume a loss of 20% between baseline and follow-up (e.g., due to mortality, relocation or inability to participate, etc.), a total of 96 participants in the intervention group and 96 in the control group will be included in the final analysis. We assume equal standard deviations in the intervention group and the control group, an alpha of 0.05 and a power of 0.80. A correction factor was applied to account for the cluster design. An average cluster size of 96 older people and an intraclass correlation coefficient of 0.02 were assumed.

The group of informal caregivers will consist of family members who are nominated by the older person (participant), and which will be involved in older people’s care for MI. In total, 50–70 informal caregivers will be involved. The group of informal caregivers will support their family members (older people) during the intervention.

The group of health professionals will be specifically selected from public administrations and local and regional healthcare providers. The group will include general practitioners and nurses from the Community Health Centre of Primorje-Gorski Kotar County as well as cardiologists from the Clinical Hospital Centre Rijeka (26) and Thalassotherapia Opatija.

For all target groups that will be invited to participate in the study, only those who sign the informed consent will participate in the study.

### 2.3 Training activities

After recruitment and before the start of the implementation phase, the research team of the Department of Social Medicine and Epidemiology of the Faculty of Medicine of the University of Rijeka will organize an initial training for health and social care professionals who will participate in the intervention phase. The training will consist of several modules that will cover the following areas:

Value-based approach, concepts of integrated care, clinical outcomes such as Patient-Reported Outcome Measures (PROMs) and Patient-Reported Experience Measures (PREMs), shared decision-making process, evaluation outcomes, the ValueCare IT ecosystem (mobile application, devices, web dashboard) and its features (goal setting and ways of interaction between users), the structure of the Integrated Care Pathway, the added value of the ValueCare model for health and social care organizations and other stakeholders, the cost-effectiveness and outcomes of VBHC implementation, and the sustainability for further permanent adoption (presenting the evidence needed to make informed decisions about adopting the ValueCare model). In addition, the modules will also address the techniques that should be used in face-to-face relationships with older people and caregivers to improve the motivation required to participate in a Pilot experience and use the IT solutions that will be deployed.

Regarding the training of older people and informal caregivers, digital skills will be the main learning objective so that older people can continue to use the devices and their versions over time, especially the devices foreseen in the project. During the training, a practical exercise on the use of the mobile application and the web dashboard will be organized. The older people will learn how to use the main features of the application, such as the Virtual Coach and access to educational materials. On the other hand, the informal caregivers will get a practical insight into the web dashboard that allows monitoring the progress of older people. This approach will certainly provide a sense of trust and reliability that is essential for transparent and strong user engagement over time.

### 2.4 Interventional methods

A total of 120 older people who had MI and completed rehabilitation in the city of Rijeka and its surroundings will participate in the intervention phase within a pre-post-controlled design research study (27). In addition, their informal caregivers, i.e., family members (50–70) and healthcare professionals (28–38), will be involved in the intervention phase to provide continuous support and monitoring to the participants.

After the intervention participants complete the baseline questionnaires (T0), an initial meeting with the healthcare professionals/cardiologists will be organized. During the first meeting, the healthcare provider and the older person will discuss the results of the questionnaire and set a personalized, value-based care plan that will be periodically reviewed as part of a shared decision-making process, a collaborative approach in which patients, working together with the healthcare professional, are encouraged to consider the treatment options available and the likely benefits and harms of each option, communicate their preferences and help choose the best course of action (39).

During the 12-month intervention period, each participant will have access to the ValueCare mobile application (Vodafone Innovus, Athens, Greece) (40), which will be based on the Virtual Coach developed by Fondazione Bruno Kessler, Trento, Italy (28). The content of the mobile application will be based on the patients’ needs and values and will be defined through the co-design activities.

In addition to the ValueCare mobile application, which will be constantly synchronized with the Vida24 ValueCare web platform, participants will also receive smartwatches, which will monitor and track heart rate, number of steps, distance covered during the day, calories burned, number of inactive hours and sleep patterns. These multisource data will constantly be uploaded to the cloud service and visible on the Vida24 ValueCare web platform, providing healthcare professionals real-time insight into the physical activity patterns of intervention participants.

Informal caregivers and healthcare professionals will play an active role during the intervention phase. Informal caregivers will have limited access to the Vida24 ValueCare web platform to gain insight into the progress of intervention phase participants. On the other hand, healthcare professionals will play several roles during the intervention period. First, they will participate in the first consultation meeting with the participants and develop the personalized value-based care plan. During the intervention, they will have full access to the web platform to evaluate the progress of their participants, to redefine the goals set up at the initial meeting via a shared decision-making process with the participants and to communicate with them. Healthcare professionals will also participate in additional health parameter measurements at the Public Health Laboratory.

To enhance the intervention phase, the Public Health Lab at the Department of Social Medicine and Epidemiology of the Faculty of Medicine, University of Rijeka, will be established. The participants will be examined every 3 months during the intervention phase, and additional analyses of health parameters such as height, weight, visceral fat (%), total body fat (%), skeletal muscle (%), body mass index (BMI), resting metabolism (kcal), blood pressure (mmHg), heart rate (b/min), oxygen saturation (%), subcutaneous fat (mm) (digital caliper), left- and right-hand dynamometry (kg), ECG, spirometry and blood sugar level (mmol/L) will be performed. These analyses will broaden the personalized care approach and provide opportunities for healthcare professionals to better adapt the needed care pathway for each participant.

### 2.5 Outcome assessment and statistical analysis

After they formally join the study, the intervention phase study participants will complete the baseline questionnaire (T0) to evaluate their frailty (Tilburg Frailty Indicator) (29), falls (Visual Analog Scale for Fear of Falling) (30), medication adherence (Medication Risk Questionnaire – MRQ-10) (31), lifestyle (BMI, smoking status, consumption of alcohol, physical activity, nutrition) (18, 32–34), daily living activities (10-item Barthel Index, which was modified) (35), physical and mental health-related quality of life (PROMIS-10) (36), comorbidities (18), loneliness (UCLA 3-Item Loneliness Scale) (37), healthcare utilization (SMRC Healthcare Utilization questionnaire) (38) and sociodemographic data (age, gender, education level, type of household income, net monthly household income, marital status and household composition). The baseline questionnaire was developed by the International Consortium for Outcome Measurement (ICHOM) as a standard for older people. The included informal caregivers and health and social care professionals will also complete baseline questionnaires. The informal caregivers’ questionnaires will evaluate their physical and mental health-related quality of life (PROMIS-10) (36), burden of informal care (iMTA Valuation of Informal Care Questionnaire – iVICQ and 4-item Zarit Burden Interview) (41, 42), level of autonomy (Adult Social Care Outcomes Toolkit) (43) and sociodemographic data. Health and social care professionals will answer questions regarding their job satisfaction (a short form of the Minnesota Satisfaction Questionnaire) (44), work-related burnout (the Copenhagen Burnout Inventory) (45), working conditions (the Culture of Care Barometer tool) (46) and sociodemographic data (age, gender, education level, occupation, net monthly household income, marital status, and household composition). The questionnaires could be completed via paper forms or on personal computers (PCs), laptops and tablets depending on target group preferences (Table 1).

**Table 1:** Timeline of the enrolment, interventions, and assessments at the Rijeka pilot site (SPIRIT figure).

	Study period					
Timepoint	Preintervention	Baseline	Intervention	Postintervention	Follow-up	Ending
	*-T0*	*T0*	*M1-M12*	*T1*	*M13-M18*	*T2*
Enrolment						
Ethical Approval	X					
Stakeholders Engagement	X					
Co-design activities	X					
Informed Consent	X					
Recruitment of participants	X					
Interventions						
ValueCare approach						
Usual care provided by the healthcare system						
Assessments						
Sociodemographic characteristics of the participants		X		X		X
Tilburg Frailty Indicator		X		X		X
Visual Analog Scale for Fear of Falling		X		X		X
Medication Risk Questionnaire		X		X		X
Lifestyle (BMI, smoking status, consumption of alcohol, physical activity, nutrition)		X		X		X
10-item Barthel Index		X		X		X
PROMIS-10		X		X		X
UCLA 3-Item Loneliness Scale		X		X		X
SMRC Healthcare Utilization questionnaire		X		X		X
iMTA Valuation of Informal Care Questionnaire – iVICQ		X		X		X
4-item Zarit Burden Interview		X		X		X
Adult Social Care Outcomes Toolkit		X		X		X
Minnesota Satisfaction Questionnaire		X		X		X
Copenhagen Burnout Inventory		X		X		X
Culture of Care Barometer tool		X		X		X

The questionnaire will be inputted through the web based GemsTracker (GEneric Medical Survey Tracker, Erasmus MC, Rotterdam, The Netherlands) (47) system, which will enable automatic evaluation via a predefined data scoring framework and data extraction in portable document format (PDF) form. Healthcare professionals will receive the questionnaire results on a pilot-specific cloud-based Vida24 ValueCare web platform (Vidavo S.A., Thessaloniki, Greece) (48).

After the one-year intervention and six-month follow-up period, all target groups who participated in the project will complete the above-mentioned questionnaires again (T1 and T2) in order to evaluate the values-based approach in its entirety. The categorical variable data will be presented as frequencies (n) and relative frequencies (%) and will be compared with appropriate test data. Depending on the distribution of the data and the results of the Kolmogorov–Smirnov test, parametric or nonparametric statistical tests will be used to assess changes before and after the intervention and at the T2 follow-up time point. A *p* value of 0.05 will be considered to indicate statistical significance.

## 3 Discussion

Value-based healthcare (VBHC) is an approach that aims to provide quality care by considering the value of patients and providers. One of the first projects to test the value-based methodology will be the ValueCare Horizon 2020 project. There are several critical elements to the successful implementation of the VBHC approach, including stakeholder engagement in recruiting participants and motivating them to participate in the pilot experience. The main strength of this approach will lie in the fact that it (i) addresses older people, whose share of the total population is steadily increasing, and (ii) proposes a plan on how to relieve the burden on the healthcare system. The second fact is closely related to the first, as the pressure on the healthcare system is expected to increase due to the larger proportion of older people in the total population. There are also advantages that arise from the fact that the approach used here is value-based, which includes the following: personalized care, shared decision-making with integration of care into the digital solution, financial and nonfinancial benefits for the patient, increased efficiency of care providers and consequently a healthier population with lower healthcare expenditure (9).

With greater satisfaction and quality of care leading to an improvement in the quality of life and bringing benefits to the patient, there is also evidence of a reduction in health expenditure (49). However, as pensions are significantly lower than the monthly salaries of the economically active population, this will have an even greater impact on this population. In addition, cost savings in one area of the health system may result in budgetary allocations to some areas that were previously underfunded. Any approach that has cost reductions as one of its main objectives will benefit older people even more.

An important part of the study included so-called “informal” (non-professional) caregivers. Using a model such as this will facilitate their involvement and make them more productive, as they will also have limited insight into the progress of the intervention. Greater involvement of informal caregivers will also contribute to some of the benefits usually associated with value-based care. For the patient, there are some financial benefits, as less direct contact with professionals is needed. More importantly, however, it will greatly reduce the workload of medical staff (professional caregivers) and allow them to spend more time with patients, as well as with informal caregivers, for the purpose of improving health outcomes and reducing the burden of informal care.

In the previous section, broad outlines of the activities were proposed. During implementation, it is important to keep in mind several key issues (or several components of a key issue-human factors). The project is designed to help older people with health problems, with a strategy based on lifestyle change, value-based care, and a personalized multidisciplinary approach. Clearly, all these areas require a certain level of personal commitment and willpower. This means that human factors play a crucial role in the successful implementation of interventions.

Testing the value-based methodology in community-dwelling patients who have suffered MI could lead to significant findings on improving health outcomes. In addition to the Rijeka pilot site, six additional pilot cities in six different European countries will provide evidence for the value-based methodology in different settings, cultures and healthcare systems. The personalized approach, based on shared decision-making using the ICHOM older person questionnaire and measurements, is a valuable tool for identifying patient progress over time, considering patient values (such as quality of life, loneliness, frailty, etc.). Digital technology will enable health professionals to capture different sets of data, share it within the health and social care system and even make decisions based on patient-reported outcome measures (PROMs). The role of custom-built technology is not only to integrate care but also to update patient data more regularly, patient engagement and clinical research. On the other hand, there is a potential risk in terms of patient safety and privacy that needs to be considered. The model described here proposes a synthesis of a unified digital solution, professional help, and the help of informal caregivers combined with the personal engagement of older people. This value-based approach should provide all the benefits associated with value-based care, i.e., less money spent (for patients), more time for patients (for healthcare professionals), a generally healthier population and a more efficient healthcare system. Considering all this, the approach is very promising and, with evidence-based development, should achieve all its goals.

The value-based approach described previously will offer personalized care for older people who suffer MI based on a shared decision-making process with healthcare professionals and informal caregivers. This approach will be developed through co-design activities that will be organized before the start of the implementation. During these activities, feedback from all relevant stakeholders (older people, informal caregivers, health and social care professionals, IT experts, managers, and exploitation stakeholders) will be considered and incorporated in the development of the personalized value-based approach supported by a robust, secure, and scalable digital solution that will consist of two parts: the ValueCare Vida24 web platform and the ValueCare mobile application. The platform will be used by healthcare professionals and informal caregivers to track the progress of older people in real time, and the mobile application, together with the smartwatch, which is based on Virtual Coach, will support the intervention participants in enhancing their overall health and quality of life and potentially reducing healthcare utilization and system expenditures.

## 4 Ethics and dissemination

This study received ethical approval from the Ethical Committee for Biomedical Research of the Faculty of Medicine, University of University of Rijeka, Croatia (class: 003–08/20–01/53; registry number: 2170-24-09-8-20-2 and class: 003–08/21–01/48; registry number: 2170-24-04-3-21-11) as well as from Ethics Committee of the Community Health Centre of Primorje-Gorski Kotar County, Rijeka, Croatia (registry number: 01–43/1–2-22). Informed consent will be collected in paper format. Any target group participants will be able to stop their participation at any time during the study without disclosing reasons for withdrawing.

All included project partners will ensure that throughout the project and, in particular, during the design of the IT solution and in the pilot implementation and evaluation, all ethical, security and data protection issues will be satisfactorily approached and dealt with. In this sense, the following specific objectives will be followed: ensuring that partners respond to the ethics and research integrity principles agreed in the Grant Agreement, ensuring the scientific and ethical standards of the intervention in each pilot site through the development of protocols, documents (i.e., informed consent forms) as well as monitoring of the whole process, guaranteeing that data privacy and protection is respected along the project implementation, especially in the cocreation, pilot and dissemination activities, ensuring trust of users through the ValueCare approach and digital solution in relation to the data access, protection and sharing, promoting a fair, open and secure digital environment along the project implementation and forward and monitoring secure and efficient sharing and processing of data and information gathered in pilots (access, protection, sharing, processing and storage).

During the implementation phase, Rijeka pilot site will constantly assess ethical issues in order to protect the rights, safety and well-being of participants within the IT solution and questionnaires. The research team will also analyze the patient data safety within the IT tool developed by the technical partners.

The main objectives for the dissemination activities within Rijeka pilot site will be directed to identify stakeholder groups and their dissemination needs, facilitate the acceptance and utilization by end users of the ValueCare approach and IT solutions, contribute to the advancement of outcome-based indicators for integrated care, inform end users (older people, health and social professionals, managers and policy makers) about the potential benefits of the value-based approach in integrated care in social and economic terms, provide information to the whole community about the value-based care approach, ensure dissemination activities cover both quantitative and qualitative outcomes which facilitate and support the impact of the project and to liaise with other ongoing EU projects in order to realize synergies and to widely exploit ValueCare’s outcomes, findings and lessons learned.
